# Sphenoclival Intraosseous Lipoma: A Typical Lesion at an Atypical Location

**DOI:** 10.7759/cureus.21732

**Published:** 2022-01-30

**Authors:** Sachin Khanduri, Saif Malik, Nazia Khan, Sonal Kaushik, Monika Panwar

**Affiliations:** 1 Radiology, Era's Lucknow Medical College and Hospital, Lucknow, IND; 2 Radiodiagnosis, Era's Lucknow Medical College and Hospital, Lucknow, IND

**Keywords:** sphenoclival region, lipoma, computed tomography, magnetic resonance imaging, intraosseous lipoma

## Abstract

In this report, we present the case of a rare tumor in the sphenoclival region and discuss the potential pitfalls in its diagnosis and management. Intraosseous lipoma is a rare benign tumor, mostly accounting for 0.1% of all bone tumors. The disease is usually asymptomatic and mainly involves the hips, vertebrae, ribs, and metaphysis of the long bones. However, the intraosseous lipoma of the skull is less common, especially with few cases having been reported to involve the sphenoid bone in the literature. We present a rare case of sphenoclival intraosseous lipoma in a 28-year-old female who presented with a history of chronic headache. A non-contrast computed tomography (NCCT) was ordered, which revealed a deviated nasal septum with thickening of bilateral ethmoidal sinuses with mastoiditis and a well-defined fat-containing intraosseous lesion in the clivus with a mean HU~ of -32 with few septations within. The risk of malignant transformation in intraosseous lipoma is very low. The differential diagnosis of intraosseous lipoma includes end stage of infection, infarct lesions, intraosseous meningioma, angiolipoma, and myxofibrous tumors.

## Introduction

Intraosseous lipoma is a very rare and usually benign tumor of flat bones. However, the localization in skull bones is described in sporadic cases. Identification of fat-containing lesions as well as dystrophic calcification and cyst formation is now facilitated with the advent of computed tomography (CT) and magnetic resonance imaging (MRI) [1,2]. Lipoma originates from mesenchymal tissue and might resemble other benign tumors. Lipoma has characteristic CT and MR features [3]. Thus, accurate identification of intraosseous lipoma is essential to avoid surgery. Intraosseous lipoma is a rare variant of lipoma, and was first reported in 1880 [4]. Involvement of the skull is even rarer. We present a case of an intraosseous lipoma in the sphenoclival region. This site is an extremely rare location for such a tumor, and to our knowledge, only a limited number of such cases have been reported previously.

## Case presentation

**Figure 1:**
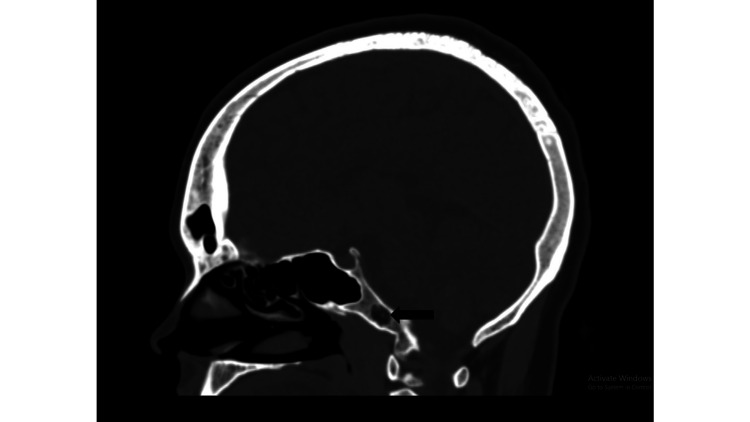
NCCT image of the skull (sagittal section) demonstrating intraosseous lipoma at the clivus (black arrow). NCCT, non-contrast computed tomography

**Figure 2:**
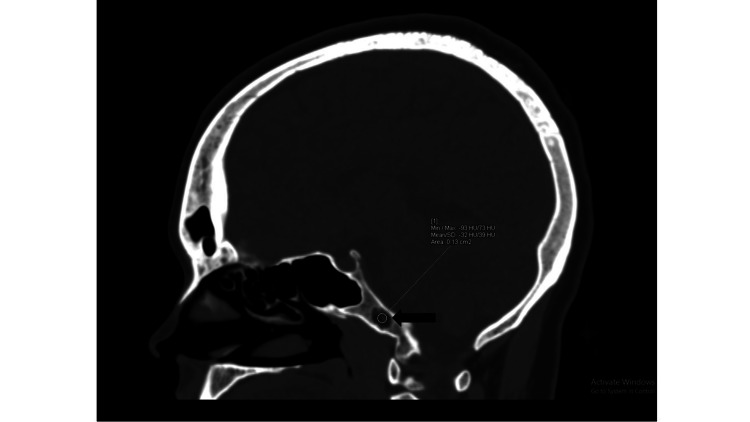
NCCT image of the skull (sagittal section) demonstrating intraosseous lipoma at the clivus (black arrow) with ROI placement revealing fatty attenuation of the lesion. NCCT, non-contrast computed tomography; ROI, region of interest

**Figure 3:**
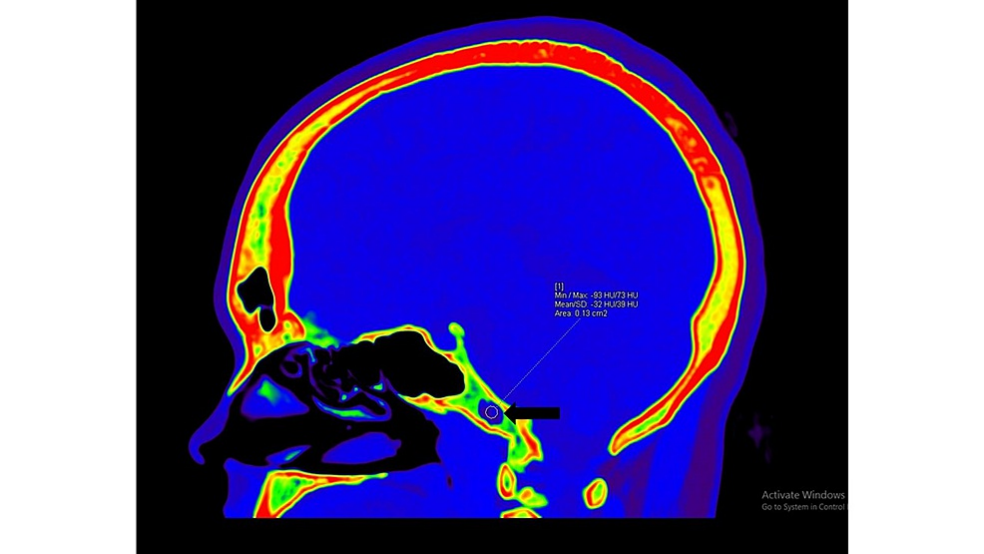
DECT image of the skull (sagittal section) demonstrating intraosseous lipoma at the clivus (black arrow) with ROI placement revealing fatty attenuation of the lesion. DECT, dual-energy computed tomography; ROI, region of interest

A 28-year-old female presented with a history of chronic headache. A non-contrast computed tomography (NCCT) of the head was ordered due to ongoing symptoms. On NCCT, we found deviated nasal septum with convexity toward the left side with a nasal spur measuring approximately 4 mm, as well as contralateral inferior turbinate hypertrophy, mastoiditis (opacification of the right mastoid air cell was noted), ethmoidal sinusitis (mucosal thickening of bilateral ethmoid sinuses), and an osteolytic lesion in the clivus with a mean HU~ of -32 with few septations within (Figures 1-3). A subsequent MRI was recommended for further evaluation. On MRI, the lesion was fairly well-circumscribed with increased signal intensity on both T1- and T2-weighted images, most consistent with intraosseous lipoma.

## Discussion

Intraosseous lipoma is a rare benign tumor, accounting for approximately 0.1% of all primary bone tumors. It usually occurs in the calcaneus, inter-trochanteric, and sub-trochanteric regions of the femur, pelvis, and flat bones. Campbell et al. [1] found that lipoma occurs most frequently in the calcaneus, whereas Milgram [5] concluded that lipoma occurs most commonly in metaphysis of the long bone. Thus, its prevalence might be underestimated. Typical benign radiographic manifestations of intraosseous lipoma may preclude further evaluation with MRI or CT. The largest case study of 66 cases was performed by Milgram [5], and after examination he divided the intraosseous lipoma into three stages (Table 1).

**Table 1:** Three stages of intraosseous lipoma as described by Milgram. [5]

Stages of Intraosseous lipoma	X-ray findings	CT findings	MRI findings
Stage 1	The lesion is radiolucent with surrounding rim of sclerosis.	The lesion demonstrates bone expansion with resorption of bony trabeculae.	The lesion demonstrates signal intensity similar to subcutaneous fat. The peripheral sclerosis exhibits a rim of low intensity on both T1- and T2- weighted sequences.
Stage 2	The lesion appears radiolucent and expansile. It shows a mixed radiolucent and sclerotic mass, representing viable fat and areas of calcification or fat necrosis, respectively.	The lesion demonstrates areas of fat attenuation corresponding to subcutaneous fat with areas of necrosis or calcification.	The lesion demonstrates circumferential rim and fat of decreased signal intensity on both T1- and T2-weighted images. Central hypointense area (low intensity on T1- and T2-weighted images) represents central calcification.
Stage 3	The lesion has thick sclerotic borders and appears denser resulting from calcification and necrosis of fat.	Lesion demonstrate peripheral rim of fat, helping it to eliminate other condition considered in differential diagnosis, and there will be calcification, fat necrosis, and cyst formation due to fat necrosis.	Peripheral rim of fat with central calcification, having low signal intensity on both T1- and T2-weighted images. Areas of fat necrosis show variable signal intensity on T1-weighted image and high signal intensity on T2-weighted image.

Recent reports revealed that sphenoid localization of intraosseous lipoma is rare [6]. Skull location of lipoma is estimated at around 4% of all intraosseous lipomas, but the incidence of the sphenoid localization has not yet been established [1,6]. Noninvasive follow-up has been proposed in case of intraosseous lipoma due to a low possibility of transforming into malignant forms, a relatively precise diagnosis based on cross-sectional imaging and sometimes spontaneous involution of the lesion [7].

Our patient has non-specific symptoms such as headache and elevated cerebrospinal fluid (CSF) protein levels. However, the relation of these symptoms to lipoma seems to be coincidental.

Intracranial intraosseous lipomas are mostly diagnosed incidentally. Treatment of intraosseous lipoma is usually conservative with observation and scanning of the lesion being recommended every six to eight months.

## Conclusions

Intracranial intraosseous lipomas are very rare bone tumors, specifically in the sphenoclival region. The differential diagnosis of intraosseous lipoma is broad and includes end stage of infection, infarct lesions, intraosseous meningioma, angiolipoma, and myxofibrous tumors. A direct diagnosis can be made using essential features such as measuring the attenuation of the lesion on CT and by fat saturation pulses on MRI. In addition to the accurate diagnosis of intraosseous lipomas, cross-sectional imaging can also be used to accurately and consistently stage these lesions.
